# Exosomes derived from mesenchymal stem cells enhance radiotherapy-induced cell death in tumor and metastatic tumor foci

**DOI:** 10.1186/s12943-018-0867-0

**Published:** 2018-08-15

**Authors:** Virgínea de Araujo Farias, Francisco O’Valle, Santiago Serrano-Saenz, Per Anderson, Eduardo Andrés, Jesús López-Peñalver, Isabel Tovar, Ana Nieto, Ana Santos, Francisco Martín, José Expósito, F. Javier Oliver, José Mariano Ruiz de Almodóvar

**Affiliations:** 1 Instituto Universitario de Investigación en Biopatología y Medicina Regenerativa, Centro de Investigación Biomédica, PTS Granada and CIBERONC (Instituto de Salud Carlos III), 18016 Granada, Spain; 2 Instituto de Parasitología y Biomedicina “López Neyra”, Consejo Superior de Investigaciones Científicas, PTS Granada, 18016 and CIBERONC (Instituto de Salud Carlos III), Granada, Spain; 3 Departamento de Anatomía Patológica, Facultad de Medicina, Universidad de Granada, PTS Granada, 18016 Granada, Spain; 4 GENYO, Centre for Genomics and Oncological Research, Pfizer/Universidad de Granada/Junta de Andalucía, PTS Granada, 18016 Granada, Spain; 5 Unidad de radiología experimental, Centro de Instrumentación Científica, Centro de Investigación Biomédica, Universidad de Granada, PTS Granada, 18016 Granada, Spain; 6 Complejo Hospitalario de Granada, Servicio Andaluz de Salud, PTS Granada, 18016 Granada, Spain; 7 Unidad de experimentación animal, Centro de Instrumentación Científica, Centro de Investigación Biomédica, Universidad de Granada, PTS Granada, 18016 Granada, Spain; 8 Unidad de microscopia, Centro de Instrumentación Científica, Centro de Investigación Biomédica, Universidad de Granada, PTS Granada, 18016 Granada, Spain

**Keywords:** Experimental radiotherapy, Bystander effect, Abscopal effect, Mesenchymal stem cells, Cell therapy, Metastasis spread, Proteomic analysis, Annexin A1, Melanoma xenograft

## Abstract

**Background:**

We have recently shown that radiotherapy may not only be a successful local and regional treatment but, when combined with MSCs, may also be a novel systemic cancer therapy. This study aimed to investigate the role of exosomes derived from irradiated MSCs in the delay of tumor growth and metastasis after treatment with MSC + radiotherapy (RT).

**Methods:**

We have measured tumor growth and metastasis formation, of subcutaneous human melanoma A375 xenografts on NOD/SCID-gamma mice, and the response of tumors to treatment with radiotherapy (2 Gy), mesenchymal cells (MSC), mesenchymal cells plus radiotherapy, and without any treatment. Using proteomic analysis, we studied the cargo of the exosomes released by the MSC treated with 2 Gy, compared with the cargo of exosomes released by MSC without treatment.

**Results:**

The tumor cell loss rates found after treatment with the combination of MSC and RT and for exclusive RT, were: 44.4% % and 12,1%, respectively. Concomitant and adjuvant use of RT and MSC, increased the mice surviving time 22,5% in this group, with regard to the group of mice treated with exclusive RT and in a 45,3% respect control group. Moreover, the number of metastatic foci found in the internal organs of the mice treated with MSC + RT was 60% less than the mice group treated with RT alone. We reasoned that the exosome secreted by the MSC, could be implicated in tumor growth delay and metastasis control after treatment.

**Conclusions:**

Our results show that exosomes derived form MSCs, combined with radiotherapy, are determinant in the enhancement of radiation effects observed in the control of metastatic spread of melanoma cells and suggest that exosome-derived factors could be involved in the bystander, and abscopal effects found after treatment of the tumors with RT plus MSC. Radiotherapy itself may not be systemic, although it might contribute to a systemic effect when used in combination with mesenchymal stem cells owing the ability of irradiated MSCs-derived exosomes to increase the control of tumor growth and metastasis.

**Electronic supplementary material:**

The online version of this article (10.1186/s12943-018-0867-0) contains supplementary material, which is available to authorized users.

## Introduction

Radiotherapy is a critical and inseparable component of comprehensive cancer treatment and care [1]. It is estimated that about half of cancer patients would benefit from radiotherapy for treatment of localized disease, local control, and palliation [2]. The success of RT in eradicating tumors depends on the total radiation dose being delivered accurately [3]. However, there are limits to the RT dose that can be given safely, which are imposed by the tolerance of the normal tissues surrounding the tumor [4, 5] and it is clear that the high intrinsic sensitivity of normal tissues to ionizing radiation often precludes the application of curative radiation doses [6, 7].

Cell membranes are intimately involved in the biochemical events that define cancers, and in particular, they are intensely involved in cancer metastasis [8]. In addition, the establishment of metastases also requires a complex interplay between malignant cells, normal cells, stroma, mesenchymal cells and extracellular matrix in their new microenvironments to facilitate invasion of extracellular matrix and tissue stroma and evade the defenses of the host [8–10].

Mesenchymal stem cells (MSCs) are found ubiquitously in many tissues and are not restricted to those of mesodermal origin such as bone marrow, adipose, muscle and bone [11]. MSC-based new therapies could potentially treat a wide range of conditions, such as cancer, inflammatory and degenerative disorders that have historically challenged patients and clinicians [12]. Although the use of MSCs for cancer therapy are considered as a useful tool in various studies [13], more research is necessary to understand their tumor promoting and suppressing potentials and to circumvent donor variations [13, 14].

The ability of MSCs to accumulate at tumor sites makes them extremely attractive for directed cancer therapy; moreover it has been described that the tumor-tropism of MSCs increase with radiotherapy [15]. Mesenchymal cells MSCs are recruited by tumors from both nearby and distant locations.

Cells can secrete ‘molecular machinery’ through several types of vesicular carriers that are composed of both membrane and cytosolic constituents. Cell secreted exosomes (30–100 nm extracellular vesicles) play a major role in intercellular communication due to their ability to transfer proteins and nucleic acids from one cell to another [16]. Depending on the originating cell type and cargo, exosomes may have either immunosuppressive or immuno-stimulatory effects, which have potential applications as immuno-therapies for cancer and auto-immune diseases [17]. In addition, exosomes might also have tumor-promoting or tumor-suppressor activities. Very recently, Hoshino and co-workers [18] have demonstrated that cell-tumor-derived exosomes prepare a favorable micro-environment at future metastatic sites and mediate non-random patterns of metastasis. Emerging evidence shows that exosomes are incipient mediators of cancer-host crosstalk and are involved in tumor initiation, growth, invasion and metastasis [8–10]. Tumor-secreting factors can also increase metastasis by inducing vascular leakiness, promoting the recruitment of pro-angiogenic immune cells, and influencing organotropism and it has been shown that tumor-derived exosomes uptaken by organ-specific cells prepare the pre-metastatic niche and may also facilitate organ-specific tumor metastatic behavior [18, 19]. It has also been described that thorax irradiation could facilitate the spread of surviving tumor cells and thus tumor recurrence under certain conditions [20], and that therapy with MSC protects lungs from radiation-induced injury and reduces the risk of lung metastasis [21].

Developments in understanding of tumor response and ways to modify it resulting from combination of RT with pharmaceutical agents to abrogate toxicity, represent an area of exciting research and development, which offer potential to improve the therapeutic ratio [3]. We have recently shown that the combination of MSC cell therapy plus radiotherapy in melanoma tumor-xenografts implanted in NOD/SCID-gamma-mice, significantly reduced the size of the established tumors, both in the primary-directly irradiated tumor as well as in the distant non-irradiated tumor [22].

Taking into account these antecedents and our previous studies [22, 23], in the current study we aimed to elucidate the mechanism by which mesenchymal cells counteract the pro-tumor and pre-metastatic actions of tumor cells through isolation and identification of key components in exosomes derived from irradiated MSCs. “Radiotherapy may not only be a successful local and regional treatment but, when combined with MSCs, may also be a novel systemic cancer therapy”.

## Material and methods

### Cell lines and culture

Umbilical-cord stromal stem cells (MSCs) were prepared and cultured as previously described [24, 25]. Tumor cell lines A375, G361 and MCF7 were cultured as previously described [23, 26]. All the cells were kept in a humidified incubator with 5% CO_2_ at 37 °C. The FBS utilized to prepare conditioned medium was depleted of bovine exosomes as described elsewhere [27] by ultracentrifugation of 50% (*v*/v) diluted FBS on DMEM at 100,000 ×g for 16 h at 4 °C. All the cell lines were routinely tested for mycoplasma following the manufacturer’s instructions and were found to be negative (e-MycoTM plus Mycoplasma PCR Detection Kit, Intron Biotechnology, Korea).

### Xenografts of A375, G361 and MCF7 cell lines

We implanted 1·10^6^ cells from the human cancer line G361, A375 or MCF7 into 7/9-week-old NOD/SCID-gamma (NSG) mice following the same procedure we used in our previous study [22]. Four groups of eight mice were treated with radiotherapy, MSC therapy, MSC therapy before radiotherapy, or left untreated (control). When necessary, mice were anesthesized with isoflurane or ketamine/medetomidine (41 mg and 0,5 mg per kg of animal weight, respectively) with reversal by atipamezole (1,2 mg/kg animal weight) to minimize anesthesia recovery duration. The total treatment duration was at least four weeks. After the final dose, we followed tumor size and mice weight and welfare for an additional 6–10 days before ending the experiments.

### Mice groups to study the A375 spontaneous metastatic process.

#### Radiotherapy group

One group (8 mice) with a tumor on each hind leg was anesthetized with ketamine/medetomidine and only one of the tumors was treated with a dose of 2 Gy. Ionizing radiation was delivered by X-Ray TUBE (YXLON, model Y, Tu 320-D03) as described previously [22]. The treatment was repeated once-a-week for a total of two weeks.

#### MSC therapy groups

Two groups (8 mice in each group) with tumors larger than 60 mm^3^ were treated with an intraperitoneal administration of 10^6^ MSC once-a-week for 2 successive weeks. The day after each cellular treatment, one of the groups (8 mice) was randomly selected to have one of their tumors irradiated. The other group was monitored and treated repeatedly with injections of MSC every week for 2 weeks.

#### Control group

One group (8 mice) with tumors on each leg was handled in exactly the same way as the irradiated and MSC injected mice, although the group did not receive either radiation or MSC therapy.

### Biodistribution of MSCs on tumor-bearing mice

We labelled MSCs with BrdU or with luciferase to follow their biodistribution when injected intratumorally or intraperitoneally. To label MSCs with BrdU we treated exponentially growing cells with 10 μM BrdU for 24 h before using them. By labelling the injected MSCs with BrdU we were able to identify them later on formalin-fixed paraffin-embedded sections of the tumors 24 h after the injection.

### Tumor growth measures and calculations

We monitored the tumor sizes every 2–3 days and measured two perpendicular diameters from each tumor to calculate tumor volume. The mathematical model applied for the analysis of this set of data obtained in our experimental therapeutic studies to measure the growth of tumors as a function of time, is the exponential growth. Under the conditions of the experiments, the logarithm of tumor volume increase linearly with time. For more details see our previous paper [22].

Using the individual tumor growth kinetics equation fitted for each one of the tumors, we calculated the necessary time for tumors to reach a volume of 2.00 ml (time to tumor growth) in a similar way to the concept previously described [28, 29], the values corresponding to each group allow us to assess the treatment efficiency in terms of increase of survival time in each one of the therapeutics groups studied, compared with the control group. Furthermore, from the fit of the experimental data to for the growth of tumors as a function of time, to an exponential equation we can obtain the value of the slope and, using this, the values for the duplication time (*TD*). The Extra sum-of-squares F-Test for comparing fits of different curves was made [22] using GraphPad Software.

### Histopathological and immuno-histochemical studies

At the end of the experiments, we recovered the xenografts from each study group, the complete thorax and the abdominal and pelvic organs and fixed in 10% buffered formalin for 48 h. Paraffin-embedded 4 μm sections were dewaxed, hydrated, and stained with hematoxylin-eosin. We determined the mitotic index, the necrotic areas and apoptotic cells observed outside the necrotic fields and a complete and protocolized macroscopic and microscopic study of the pelvic, abdominal and thoracic organs was done to assess possible metastasis. We studied one histological section of heart, mediastinum, spleen, pancreas; a longitudinal section of kidneys, the genital tract, a segment of the large intestine, and lymph nodes found plus all the lung lobules and five longitudinal liver sections. It was considered as different metastatic foci if there were interposed healthy parenchyma between groups of more than 10 neoplastic cells. For further details on Exosomes purification, characterization and analysis, proteomic analysis, and statistical analysis see Additional file 1.

## Results

**Fig. 1:**
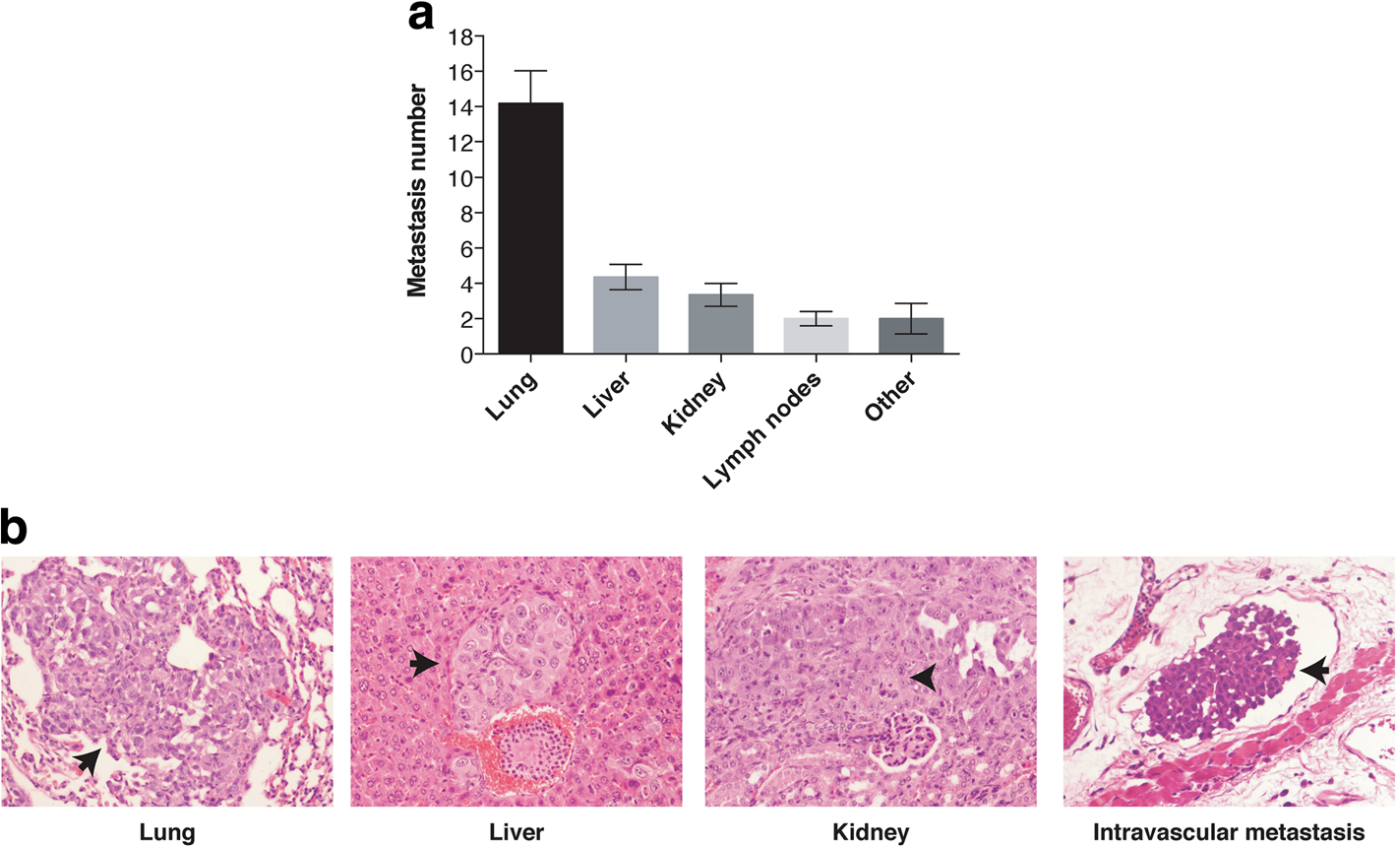
**a** Distribution of A375 xenografts’ micro-metastasis in the internal organs of the tumor-bearing NOD/SCID-gamma mice. Results are expressed as mean value ± standard error of mean. **b** Representative photomicrographs of H&E from lungs, liver, kidney and intravascular micro-metastasis (black arrow)

Previously we have shown that MSCs increased their tumor suppressor activity when they are activated with radiotherapy. In the current study we wondered if this anti-tumor action could also be relevant in decreasing metastatic spread. To assess this effect, we implanted three different tumor cell lines, G361, A375 and MCF7, in both flanks of NOD/SCID mice to produce bilateral xenografts. Our results demonstrate that the A375 human skin-melanoma cancer cell line, when implanted as xenografts, in the NOD/SCID-gamma mice growth faster than G361 and MCF-7 cell line xenografts moreover, A375 xenografts are able to spread from its initial location to produce metastases in the internal organs of the mice, whereas, in our model, the cell lines G361 and MCF7 lack this potentiality (Additional file 1: Table S1). 60 out of the 97 mice bearing A375 xenografts showed metastatic spread (Fig. 1a). Of these mice, 59 of the 60 (98.3%) showed lung poli-metastasis, the mean number of metastatic foci on lungs being 14.2 ± 1.8. After that, the organs more frequently invaded by the tumor cells are the liver (33/60; 55.0%); the kidney (20/60; 33.3%) and the lymph nodes (4/60; 6.7%). The data suggest that the lung is the initial target of metastatic dissemination and after this step, and more slowly, tumor cells may reach the liver and/or kidney. Thus, for the rest of the study we used A375 cell line as model to evaluate the effect of radiotherapy, mesenchymal cell and MSC plus radiotherapy on the tumors (irradiated and bystander) and on the metastatic spread process.

### Biodistribution of MSC injected or infused in mice

**Fig. 2:**
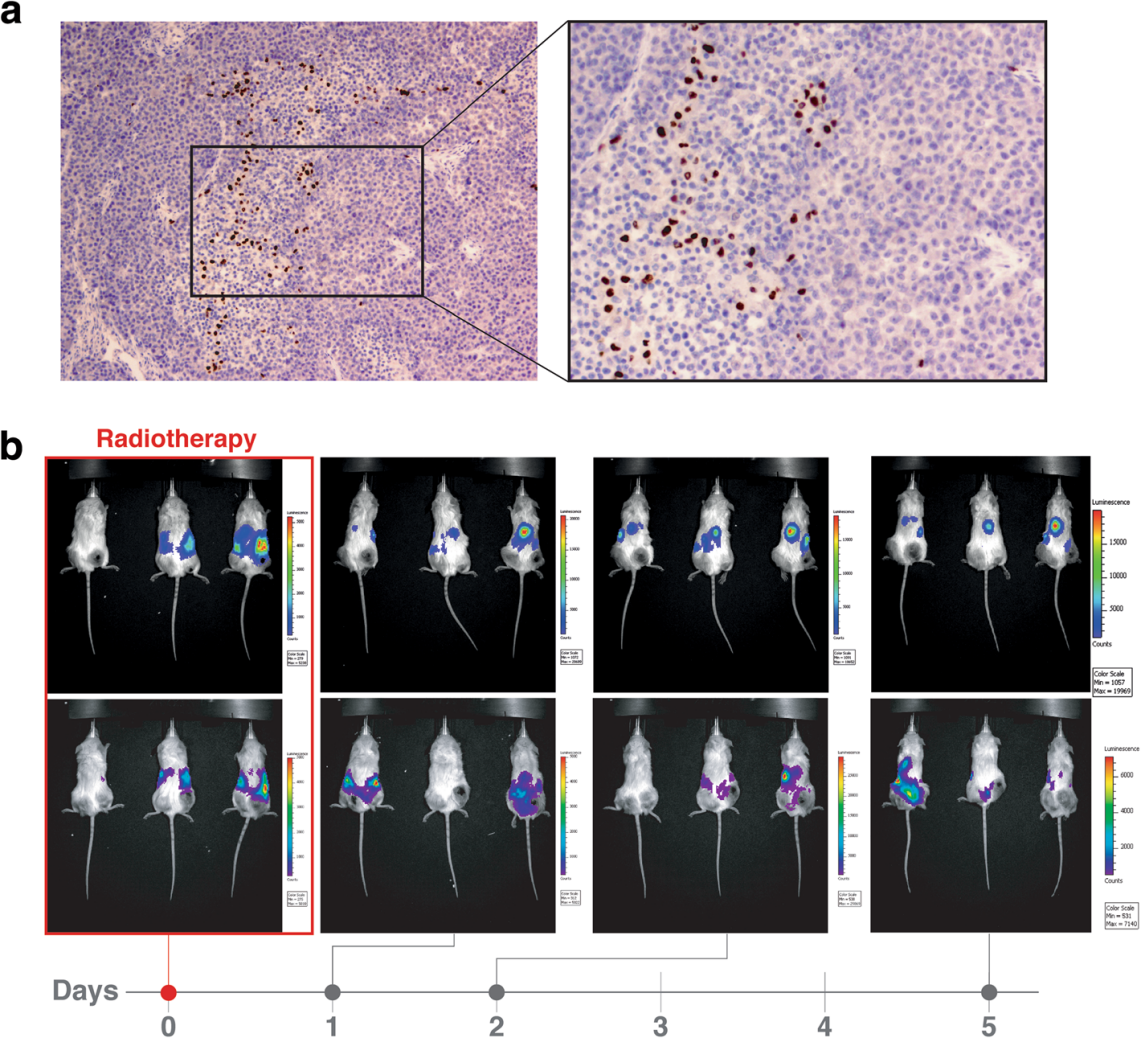
Biodistribution of MSC on tumor-bearing mice injected intratumorally or intraperitoneally. **a** Representative photomicrographs of A375 xenografts 24 after BrdU-labelled MSCs (brown) intratumoral injection. **b** Biodistribution of luciferase-labelled MSCs injected intraperitoneally on tumor-bearing NSG mice after receiving radiotherapy (right flank). The images were obtained at 0, 1, 2 and 5 days after injecting (intraperitoneally) 10 luciferase-expressing MSCs. Luciferin was injected 5 min before the images are obtained

To study the movement of cells inside the A375 xenografts we injected MSCs labeled with BrdU (10^6^ cells) intratumorally and performed the histological study 24 h post-injection (Fig. 2a). The histological study shows that injected (brown) MSCs were present inside the tumor tissue and that they localized along longitudinal trajectories whose tracing could be associated with the existence of neo-formed vessels within the xenografts. In fact the shape of the MSCs resembles that which is characteristic of normal pericytes as has been previously described: Once inside the tumor MSCs are incorporated into their stroma and could remain, as pericytes, in the environment of the vessel walls that nourish the neoplastic process [30].

We also studied the biodistribution of MSCs, genetically modified to express the luciferase gene on tumor-bearing NSG mice. Figure 2b shows images (IVIS-Lumina II) corresponding to mice with A375 tumor xenografts placed on the upper part of both hind legs. We treated the tumor on the right flank of the mice with RT (2 Gy). Right after the MSC injection, the luminescence occupies the abdominal region. At day 1 the pattern of cell distribution is different and suggests that the highest cell density is found in the central region of the mouse body and in its pulmonary and circulatory systems. At day 2, apart from the central focus, there is another region with intense bioluminescence that seems to fit to the border of the irradiated tumor. This pattern is maintained 5 days after the injection of cells.

### MSC combined with RT reduces the number of observed metastasis.

**Fig. 3:**
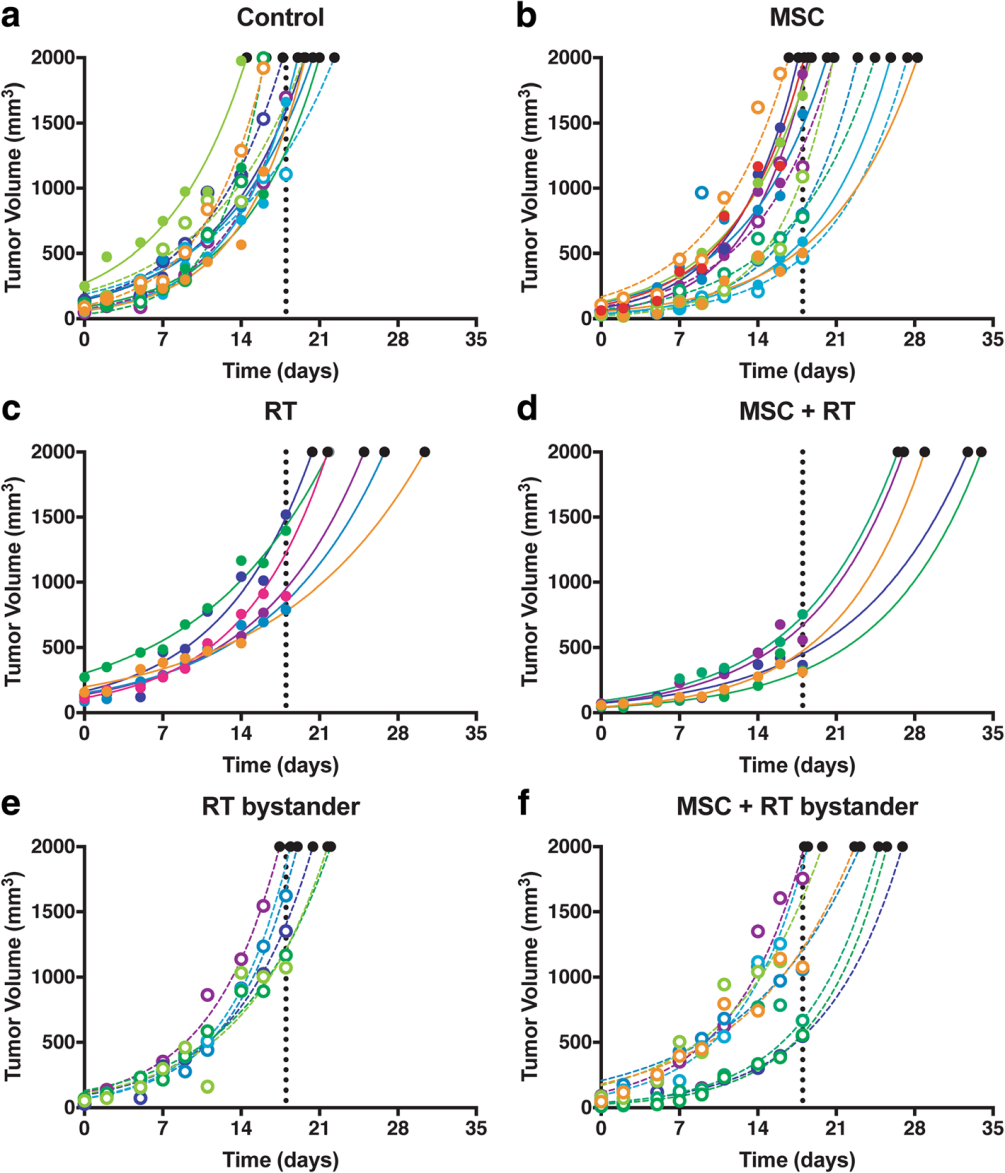
Kinetics of individual tumor growth and its treatment response to (**a**) Control, (**b**) MSC, (**c**) RT, (**d**) MSC + RT, (**e**) bystander tumor after RT and (**f**) bystander tumor after MSC + RT. Black points at the of the extrapolated curves represents the time to tumor growth (*T-t-G*) values. The different colors on the graphs represent a different animal within each group. Full dots represent the treated tumor (when applicable) and open dots represent its contralateral tumor

To further evaluate the anti-metastatic potential of MSCs combined with RT, we have carried out experiments to follow tumor-volume growth kinetics during a time-course of only 14 days. Reducing the duration of the experiment reduced the probability of a massive metastatic spread of tumor cells in almost all mice included in the study, regardless of the treatment, and allowed us to assess the differences among the groups. All the growth curves obtained either in Control as in MSC, RT and RT + MSC groups has been plotted in Fig. 3.

**Fig. 4:**
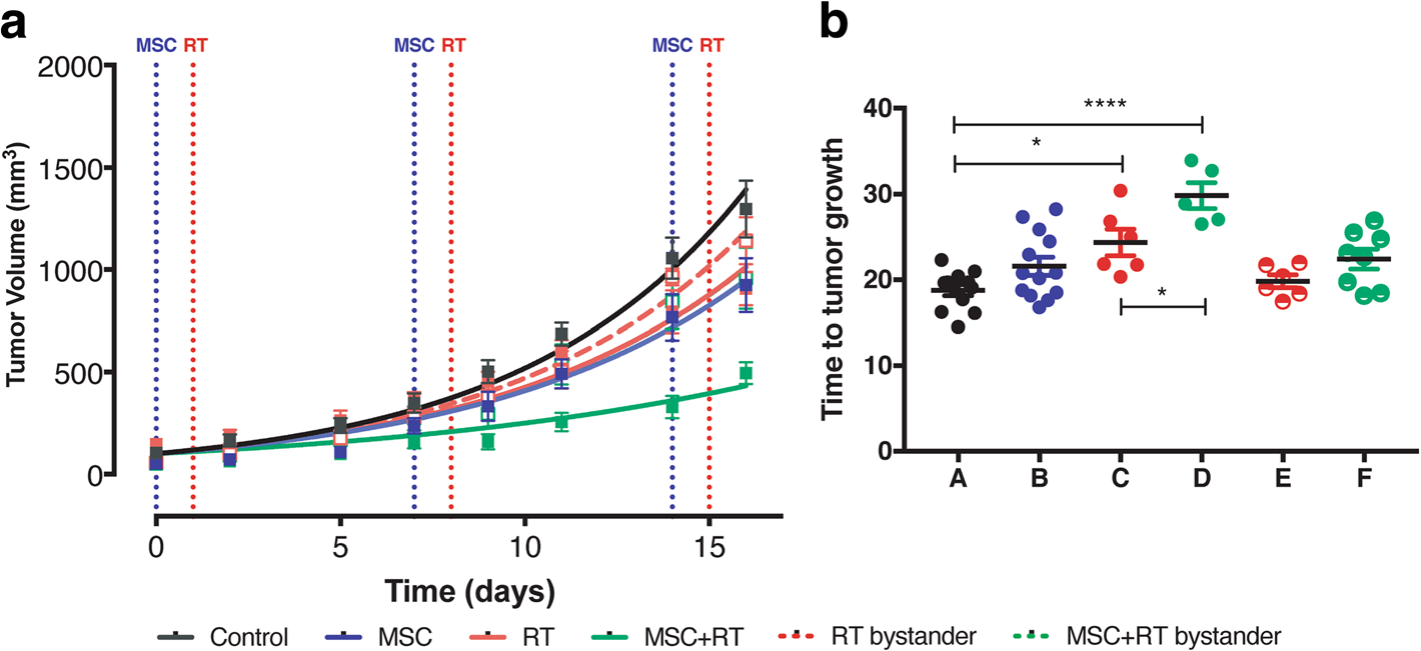
**a** Mean values of the growth kinetic curves of the xenotumors infused on NSG mice. Different curves hat different slopes (*P* < 0,0001) and the differences between RT and RT + MSC curves are also significant (P < 0,0001). **b** Extrapolated values of the time to tumor growth until a volume of 2,00 ml. The treated tumors growth slowly than un-treated and bystander tumors. The mean value of tumors with RT + MSC is significantly longer than the *T-t-G* in the group treated with RT alone

We made one key assumption in the model: Tumor growth rate is constant in the interval of time between the start of data acquisition and the end of the experiment, and treated tumors grow slower than the control tumors because their doubling times are longer. Comparing the tumor response curves in the mice treated with RT, MSC, MSC + RT or without any treatment, (Fig. 4a), we have observed an improved tumor response in the group treated with MSC + RT (green curve) compared to the groups of mice treated with RT (red curve, *P* < 0.0001) or with MSCs (blue curve, P < 0.0001), exclusively. We have shown that this mathematical model properly described the growth A375 tumors, until more than 30 days (Additional file 2: Figure S1).

The calculated values of duplication time (days) are the following: Control = 4,21; MSC = 4,93; RT: 4,79; RT + MSC = 7,57, Bys-RT = 4,48 and Bys-RT + MSC = 4,80.

Using these values, we have calculated the cell-loss rate (C_L_) that can be attributed to each treatment [22, 31]. The C_L_ for RT + MSC was: 0,44 and for RT alone 0,12. According to this concept we can state that radiotherapy inhibited tumor growth with a cell loss rate of 12.0% per day compared to tumor growth in the control group. This effect was enhanced by the addition of MSCs to the radiotherapy, with cell lost rate of 44,4% per day, leading to a mesenchymal enhancement ratio of MSC-ER = 3,7, whilst MSCs alone inhibited tumor growth with a cell loss rate of 14,5%. Assuming that the effects for each of the treatments (RT and MSC) are independent [32], we have calculated that the expected value (E) for the surviving fraction after the treatment with RT + MSC is: E = 0,75. On the other hand, the observed value for the surviving fraction after RT + MSC is O = 0,44. Using both data we have calculated the ratio O/E = 0.59, which is a strong indicator of the synergistic effect [32] between RT and MSC when they are applied together for tumor treatment in this model. These results demonstrate the potentiation of the bystander effect by the MSCs used together with radiotherapy.

To get an approach to the survival of the mice in each group, we have calculated the time-to-tumor growth (*T-t-G*), a theoretical tumor end-point-time for tumor growth [28], defined in this case as the time necessary for each tumor to reach the volume of 2,00 ml. The differences between the times necessary for the tumors from each group to reach 2,00 ml among the groups are statistically significant (Fig. 4b, P < 0.0001) and specifically the use of MSC together with RT increase the time-to-tumor growth of mice included in the therapeutic groups, with respect to the mice included in the control group. Values of time-to-tumor growth have been plotted in Fig. 4b and, interestingly, the combined treatment RT + MSC produces a clear enhancement of the radiotherapy efficacy measured as an increase in this parameter.

Our results demonstrate that the combined treatment with RT + MSC increases the surviving time of the mice included in this group by 5 days (22%) compared to the group of mice treated exclusively with RT and more than 11 days (60%) compared to the control group. Of interest is the bystander effect of the radiotherapy on the tumor of the contra-lateral side, which by itself led to an inhibition of tumor growth corresponding increase of 1 day in the *T-t-G*. Tumors from the non-irradiated flank, thus exposed to the bystander effect after RT + MSC treatment, showed a further inhibition of tumor growth increase in *T-t-G* of 3,6 days compared to the tumor growth under control conditions.

**Fig. 5:**
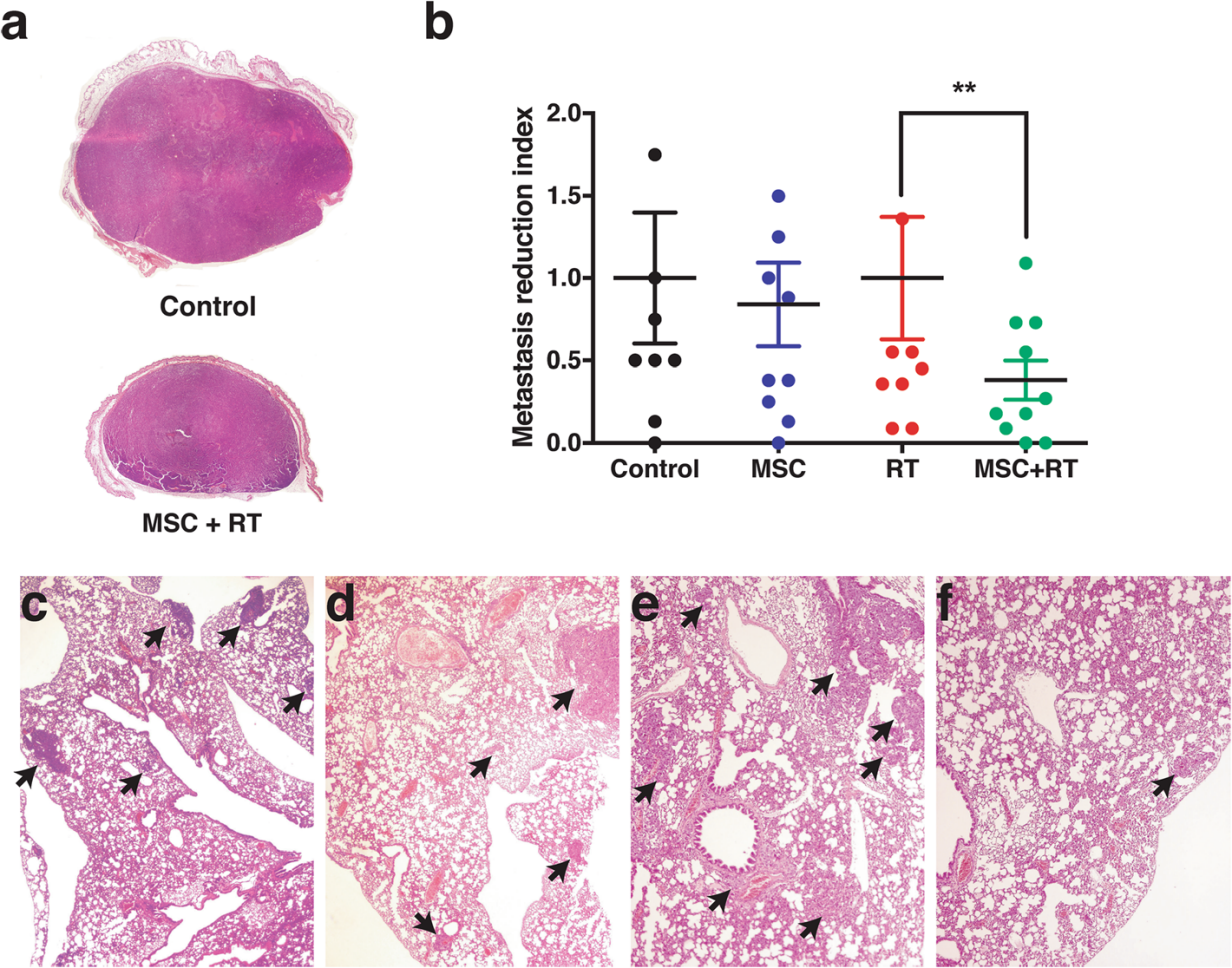
Histopathological study of the internal organs of the tumor-bearing mice treated for only 15–17 days, (**a**) Representative panoramic images of A375 human melanoma xenografts from Control and MSC + RT. **b** Comparisons between the metastasis incidence of Control vs. MSC and RT vs. MSC + RT. MSC treatment alone had no effect on the metastasis incidence index. The combined treatment of MSC + RT diminished the early spread of metastases produced by the A375 xenograft. Representative photomicrographs of H&E lung micro-metastases from (**c**) Control, (**d**) MSC, (**e**) RT and (**f**) MSC + RT treatment groups

Next, we analyzed the amount of metastasis foci present in each of the mice included in the different groups. Metastases were microscopically identified and counted to calculate their frequency. Figure 5a illustrate the difference in size between A375 xenografts respect to control and MSC + RT groups at the end of the experiment. To further quantify the inhibition of tumor foci by MSCs the number of metastasis pooled in each cohort of mice was expressed as a fraction of its respective control group. We compared: control vs. MSC and RT vs. RT + MSC. These results are summarized in Fig. 5b. The analysis of Fig. 5b suggest that infusion of MSCs accounted for a reduction of approximately 62% (*P* = 0.0020) if we compare mice treated only with RT with the group treated with RT + MSC, indicating that the combination RT + MSCs produces an important reduction in the metastatic potential of the human melanoma A375 xenografts. Figure 5c-f exemplifies the amount of micrometastasis on lungs of mice from control, MSC, RT and MSC + RT groups.

### Exosomes secreted from MSCs are quantitatively, functionally and qualitatively different from the exosomes secreted from MSCs*

**Fig. 6:**
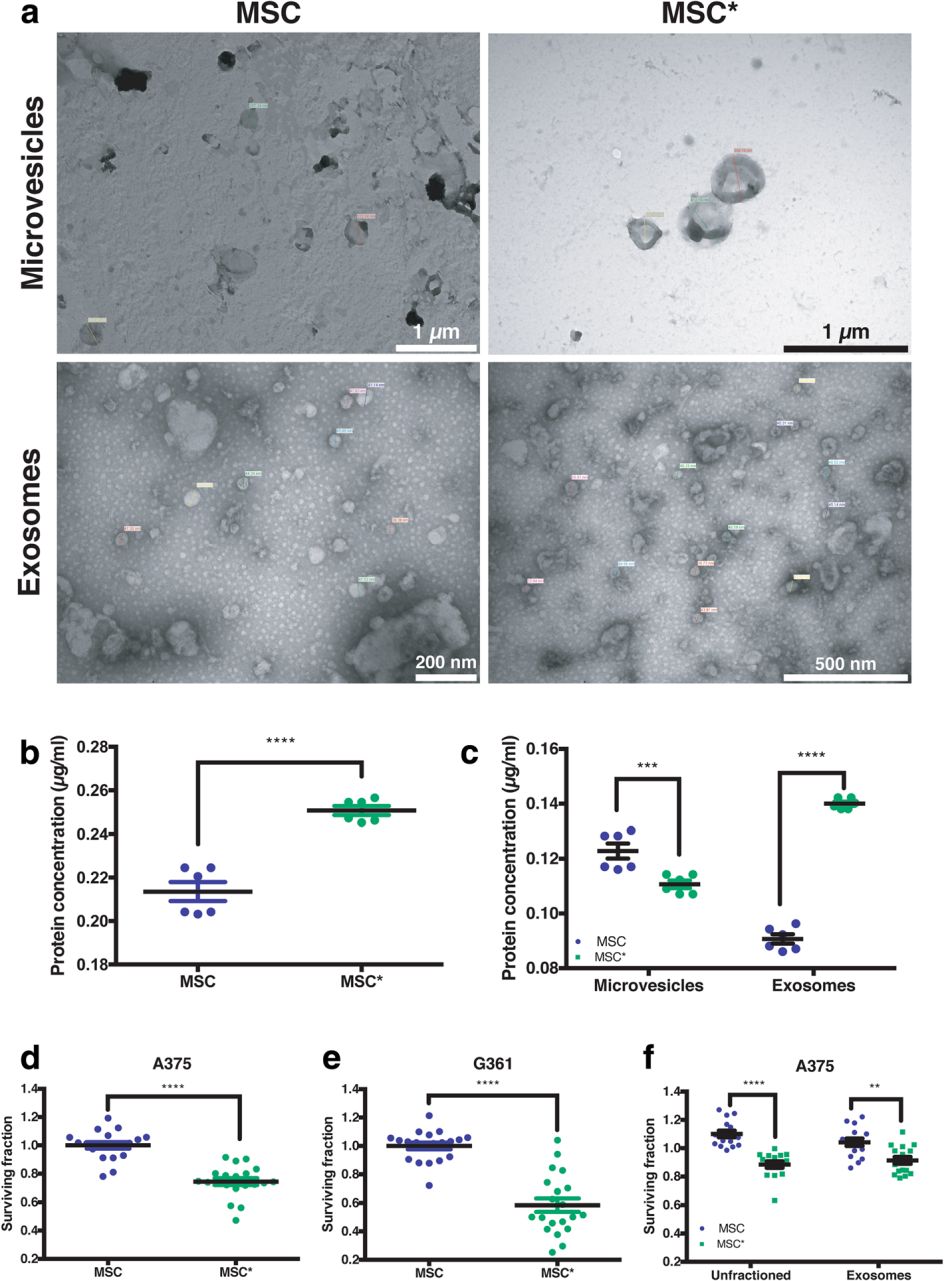
**a** Morphologic characterization of the extracellular vesicles released by MSC and MSC* precipitated by differential ultra-centrifugation. **b** Total protein concentration on the extracellular vesicles released by MSC and MSC*. **c** Protein concentration on the microvesicles and exosomes from MSC and MSC*. MSC or MSC* unfractioned conditioned medium reduced the surviving fractions of (**d**) A375 and (**e**) G361 cells. **f** Comparison between unfractioned conditioned medium from MSC and MSC* and of its exosomes on the A375 cell line. MSC conditioned medium (blue points) has been considered as the control as there is any statistical differences between MSC and growth media controls (data not shown). Differences are statistically significant between conditioned medium (*P* < 0.05) and exosomes (green points, *P* < 0.0001) from MSC and MSC*

Exosomes (Exo) and microvesicles (MV) secreted by mesenchymal cells, from both inactivated MSCs and activated MSCs*, have been quantified measuring the amount of protein present in each of the fractions from the sequential centrifugation method used to separate MV and Exo. Typical images of the Exo and MV from MSCs, obtained by transmission electronic microscopy, are summarized in Fig. 6a. The values of protein concentration in the paired experiment designed for this purpose, suggest that the treatment of MSCs with 2 Gy of low-LET ionizing radiation dose produces the activation of the irradiated cells, increasing the secretion of proteins to the culture media by the stimulated cells (Fig. 6b): MSC* = 0.251 ± 0.002 μg/ml vs. MSC = 0.214 ± 0.004 μg/ml, P = < 0.0001. The differences between proteins in MV and Exo from MSC and MSC* are statistically significant (Fig. 6b): Exo: MSC = 0.091 ± 0.002 μg/ml vs. MSC* = 0.140 ± 0.001 μg/ml, *P* < 0.0001 and MV: MSC = 0.123 ± 0.003 μg/ml vs. MSC* = 0.111 ± 0.001 μg/ml, *P* = 0.0002 (Fig. 6b). Our data demonstrate that the exosomes secretion in MSCs* increased 1.5 fold times respect to MSCs.

### Exosomes and proteins secreted by MSCs might be involved in the antitumoral effects observed

Exosomes are pivotal in facilitating intercellular communication [33]. We wonder if the exosomes produced by MSCs and MSC*, can modulate the growth of tumor cells by affecting major cellular pathways that lead to the cell death of the tumor cells, which could be the protein “cargo” contained in these exosomes [16] and whether it is possible to identify differences between the tumor-suppressor activity of exosomes obtained from MSCs and from activated MSCs*.

Figure 6d-f summarizes the results of a cell survival assay [34] adapted to measure G361 and A375 survival fraction. We compared the survival fractions of tumor cells treated with MSC or MSC* conditioned medium (Fig. 6d-e) and then compared the effect of MSC* exosomes on A375 cells (Fig. 6f). The potency index is defined as the relation between the estimated surviving fraction from MSC*-exosomes and the MSC-exosome treated cells.

**Fig. 7:**
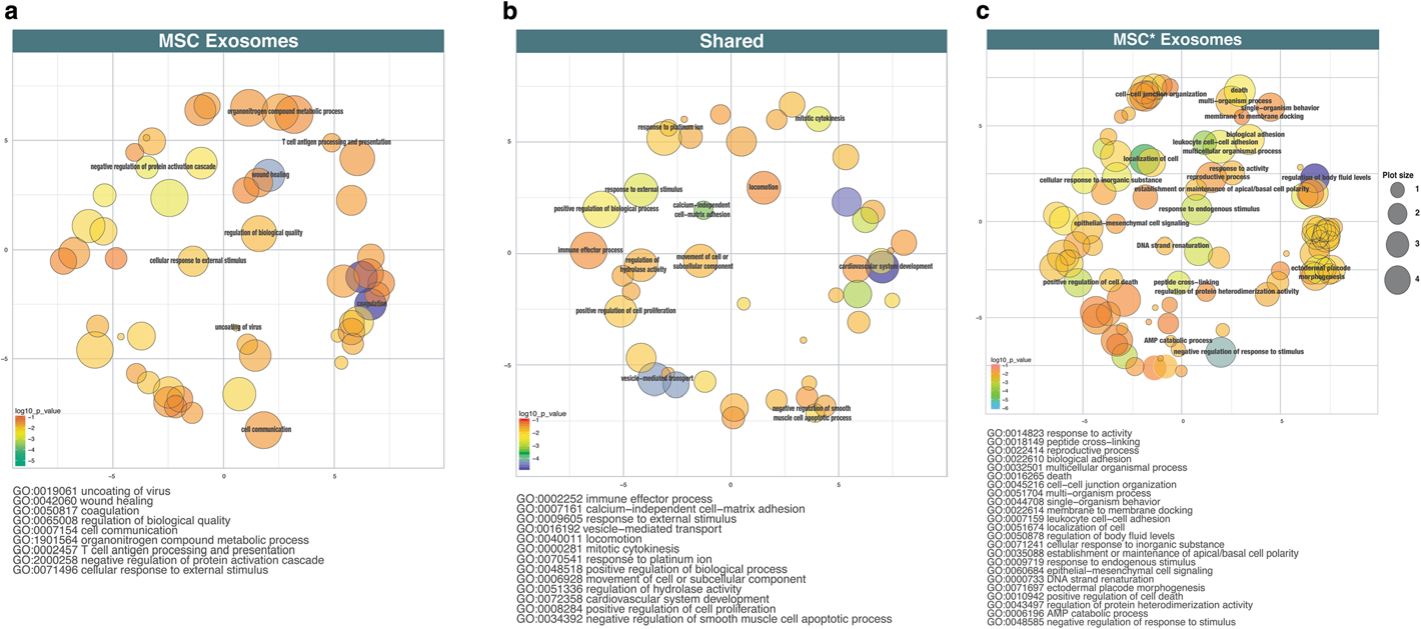
Cluster analysis of the Gene Ontology (GO) Biological Process terms from the common and unique proteins identified in the exosomes of MSC and MSC*. Terms, represented as circles, are collapsed into clusters according to semantic similarities. The labels on the graphics represent the most unique terms. Circle colors reflect the *p*-values and circle sizes the number of identified proteins within the GO term. **a** MSC exosomesc. **b** Shared exosomes between MSC and irradiated MSC (MSC*). **c** MSC* exosomes

MSC* exosomes reduce the cell survival of A375 cells (P < 0.0001) as the unfractioned conditioned media of MSC* does. This indicates that the activation of MSCs with 2 Gy increases its tumor-suppressor effect. As we found a dramatic cytoreductor effect of MSC* exosomes on the tumor cells, we have examined the protein content in these nanosized oraganelles. The results of these experiments are in Fig. 7 and in Additional file 1.

### Exosome function enrichment

To further characterize the functionality of the exosome content from MSC and MSC* we have used a bioinformatics tool aimed to identify the signaling pathways involved in different key cellular process. Significant biological process terms from REVIGO were studied in detail (Fig. 7). 15 common terms were obtained between MSC and MSC* results, 20 terms were exclusively enriched in MSC and 41 from MSC* (*p*-value < 0.01 or log10 p-value<− 2), as shown in detail in Additional file 1. According the uniqueness values, dispensability values and *p*-values, common GO terms between MSC and MSC* are related to calcium-independent cell-matrix adhesion, transport mediated by vesicles, platelet degranulation and activation and cardiovascular development (see details in Fig. 7). However, enriched terms from MSC exosomes are correlated to wound healing, coagulation, hemostasis and regulation of immune response (displayed in Fig. 7). Interestingly, MSC* analysis generated the most prominent and diverse terms in relation to the control of tumor growth, in particular the negative regulation of response to stimulus, localization of cell, leukocyte cell-cell adhesion and positive regulation of cell death (shown in Additional file 1).

## Discussion

In this paper, we present a set of preclinical therapeutic data in which we combine RT with MSC therapy. We have demonstrated that tumor cell loss induced after treatment with radiotherapy increases with the combination of RT and MSCs, reaching 51.4% per day when compared to RT alone, which was only 25.8% per day with an MSC enhancement ratio of around 2 (Additional file 2: Figure S1). These values indicate that the combination of MSC + RTs produces a synergic effect. Furthermore, we have calculated the differences in the time necessary to reach 2,00 ml of tumor volume from the different groups (Fig. 4b). Tumors treated with RT alone would need 24.4 days to reach this volume and mice treated with the combination of MSC + RT would need 29.8 days. Our results demonstrate that the concomitant and adjuvant use of RT and MSCs could represent an increase of the surviving time of the mice included in this group of around 22%, compared to the group of mice treated exclusively with RT. Moreover, the number of metastatic foci found in the internal organs of the mice treated with MSC + RTs was a 60% less than in the group of mice treated with RT alone.

The paracrine effect of MSC was first described almost two decades ago by Haynesworth and co-workers [35]. Extracellular vesicles such as exosomes are naturally released from MSCs and in our model might be responsible for the survival reduction of tumor cells in vitro. Understanding the fundamental biology underlying mesenchymal stem cell and tumor interactions has the potential to increase our knowledge of cancer initiation and progression, and also lead to novel therapeutics for cancer. Exosomes derived from mesenchymal stem cells seems to be key players at this respect. Due to their properties, MSCs may be qualified as a therapeutic tool to treat radiation-induced tissue damage [36]. Numerous studies have shown that administered either intraperitoneally or intravenously MSCs efficiently home onto tumors and metastases [37, 38]. Exosomes secreted by MSCs have been shown to contain antiapoptotic miRNAs, to promote epithelial and endothelial wound healing and angiogenesis, and to contain growth factor receptor mRNAs, known to promote wound healing and protect the intestines from experimental necrotizing enterocolitis [39]. We have demonstrated in vitro, that exosomes separated from the culture medium of MSCs are quantitatively, functionally and qualitatively different from the exosomes obtained from MSC activated cells. When we analyzed the exosome “cargo”, before and after activation with RT, we have found important differences in the proteomic content between the samples.

Our results (Fig. 7 and Additional file 1) show that there are qualitative differences between the proteins contained in the exosomes obtained from MSCs and MSCs*.

According with the GO terms obtained through a hypergeometric analysis, we found a different enrichment of terms between MSC and MSC* exosomes in different biological processes as well as in the number of pathways affected. Thus, whereas the numbers of highly significant common GO terms and MSC terms are in consonance, MSC* results generated a large variety and number of pathways altered, and it demonstrate the profound metabolic alteration that have undergone these exosomes.

Consequently, the results show that common GO terms and MSC terms are similar and related with exosome functions. Therefore, as shown in Additional file 1 the distribution of clusters is analogous and clusters representatives are associated with transport mediated by vesicles, coagulation (through platelet roles), development processes or immune response. On the contrary, as shown in Fig. 7, and Additional file 1, MSC* enriched terms are more disperse, having different and interconnected clusters with a complex biological background. Among the more representative clusters we highlight leukocyte cell-cell adhesion, cell localization, and negative regulation of responses to stimulus and cell death. Some of these proteins are key components of cell-cell or cell-matrix adhesion (Additional file 1) and includes annexin and integrins such as ANXA1, ANAX2, ITGB1, ITGA3, FN1, CTNNB1, APOH which interplay may activate exosome and leukocyte adhesion to tumor cells to limit tumor growth. The presence of annexin is very significant only in the exosomes released from MSCs*. The prototype member of this family, ANXA1, has been widely recognized as an anti-inflammatory mediator affecting migration and cellular responses of various cell types of the innate immune system [40]. Moreover, ANXA1 mRNA was tremendously up-regulated following MSCs irradiation (Additional file 2: Figure S2). Interestingly some key biological aspects of ANXA1 (potential tumor suppressor gene, ability to modulate tumor cells apoptosis induced by ionizing radiation and radiotherapeutic efficacy) deserve future studies to fully elucidate its role in the therapeutic effect of exosome derived from irradiated MSCs.

The therapeutic efficacy of transplanted MSCs actually seems to be independent of the physical proximity of the transplanted cells to damaged tissue. The number of MSCs that engraft into injured tissues may not be sufficient to account for their robust overall protective effects. Exosomes secreted by MSCs have been shown to contain anti-apoptotic miRNAs, to promote epithelial and endothelial wound healing and angiogenesis, and to contain growth factor receptor mRNAs, known to promote wound healing. Considered to be a vectorized signaling system, we believe that the exosomes released from MSCs seem to bind to specific membrane micro-domains on tumor cells, which widen the radiotherapy action, by stimulating tumor cell death thus increasing the sensitivity of cells to radiation and promoting the systemic effects. This hypothesis provides a rationale for the therapeutic efficacy of MSCs and their secreted exosomes in a wide spectrum of diseases, and also rationalizes the additional use of MSC exosomes as an adjuvant to support and complement other therapeutic modalities [11].

## Conclusions

Our results show that exosomes derived from irradiated MSCs may be a determinant factor in the enhancement of radiation effects leading to increased metastasis control. Radiotherapy itself may not be systemic, although it might contribute to a systemic effect when used in combination with mesenchymal stem cells.

## Additional files

Additional file 1: Significant biological process terms from REVIGO (Reduce + visualize gene ontology). (XLS 51 kb)

Additional file 2: **Figure S1.** (a) Tumor growth kinetics and response to radiotherapy administered twice-a-week alone or in combination with simultaneous MSC* injection. The combination of radiotherapy and MSC* reduced tumor growth rate more than radiotherapy alone did. (b) Calculated time to tumor growth (T-t-G) for each group. As a result of the reduction on tumor growth kinetics, tumors from the group receiving the combination of RT + MSC* would need more days to reach 2,0 ml. 3. What do the authors mean by MSC* + RT in Fig. S1. The notation (MSC* + RT) means: MSC*: in vitro activated (2 Gy of low-LET (lineal energy transfer) radiation) mesenchymal cells were administered intraperitoneally; RT: 2 h after MSC* injection tumors were treated locally with radiotherapy (RT, 2Gy). This combined treatment was repeated every 4 days during a total of 24 days. **Figure S2.** mRNA expression of TRAIL, DKK3 and ANXA1 by MSCs 24 and 48 h after receiving 2 Gy of radiation. The overexpression of TRAIL and DKK3 is consistent with our previous study [22], the ANXA1 overexpression is consistent with the presence of the protein form inside the MSC* exosomes. (ZIP 1301 kb)

## Abbreviations

ANXA1: Annexin1
ANXA2: Annexin 2
APOH: Apolipoprotein H
BrDU: Brdeoxyuridine
CTNNB1: Catenin beta 1
ER: Enhancement ratio
Exo: Exosomes
FBS: Fetal bovine serum
FN1: Fibronectin 1
ITGA3: Integrin A3
ITGB1: Integrin B1
MSC: Umbilical-cord stromal stem cells
MV: Microvesicles
NSG: NOD/SCID-gamma.
PCR: Polymerase chain reaction.
REViGO: Reduce and visualice gene ontology.
RT: Radiotherapy.
T-t-G: Time-to-tumor growth.

## References

[1] Atun R, Jaffray DA, Barton MB, Bray F, Baumann M, Vikram B, Hanna TP, Knaul FM, Lievens Y, Lui TY (2015). Expanding global access to radiotherapy. The Lancet Oncology.

[2] Barton MB, Jacob S, Shafiq J, Wong K, Thompson SR, Hanna TP, Delaney GP (2014). Estimating the demand for radiotherapy from the evidence: a review of changes from 2003 to 2012. Radiother Oncol..

[3] Scaife JE, Barnett GC, Noble DJ, Jena R, Thomas SJ, West CM, Burnet NG (2015). Exploiting biological and physical determinants of radiotherapy toxicity to individualize treatment. Br J Radiol.

[4] Burnet NG, Wurm R, Nyman J, Peacock JH: Normal tissue radiosensitivity--how important is it? *Clinical oncology (Royal College of Radiologists (Great Britain))* 1996, 8**:**25–34. 10.1016/s0936-6555(05)80035-4 8688357

[5] Lopez E, Guerrero R, Nunez MI, del Moral R, Villalobos M, Martinez-Galan J, Valenzuela MT, Munoz-Gamez JA, Oliver FJ, Martin-Oliva D, Ruiz de Almodovar JM (2005). early and late skin reactions to radiotherapy for breast cancer and their correlation with radiation-induced DNA damage in lymphocytes. Breast Cancer Res.

[6] Dietrich A, Koi L, Zophel K, Sihver W, Kotzerke J, Baumann M, Krause M (2015). Improving external beam radiotherapy by combination with internal irradiation. Br J Radiol.

[7] Lopez E, Nunez MI, Guerrero MR, del Moral R, de Dios LJ, del Mar RM, Valenzuela MT, Villalobos M, Ruiz de Almodovar JM (2002). breast cancer acute radiotherapy morbidity evaluated by different scoring systems. Breast Cancer Res Treat.

[8] Nicolson GL (2015). Cell membrane fluid-mosaic structure and cancer metastasis. Cancer Res.

[9] Kumar D, Gupta D, Shankar S, Srivastava RK (2015). Biomolecular characterization of exosomes released from cancer stem cells: possible implications for biomarker and treatment of cancer. Oncotarget.

[10] Zhou W, Fong MY, Min Y, Somlo G, Liu L, Palomares MR, Yu Y, Chow A, O'Connor ST, Chin AR (2014). Cancer-secreted miR-105 destroys vascular endothelial barriers to promote metastasis. Cancer Cell.

[11] Lai RC, Yeo RW, Lim SK (2015). Mesenchymal stem cell exosomes. Semin Cell Dev Biol.

[12] Prockop DJ, Prockop SE, Bertoncello I (2014). Are clinical trials with mesenchymal stem/progenitor cells too far ahead of the science? Lessons from experimental hematology. Stem Cells.

[13] Shah K (2012). Mesenchymal stem cells engineered for cancer therapy. Adv Drug Deliv Rev.

[14] Zhao Q, Gregory CA, Lee RH, Reger RL, Qin L, Hai B, Park MS, Yoon N, Clough B, McNeill E (2015). MSCs derived from iPSCs with a modified protocol are tumor-tropic but have much less potential to promote tumors than bone marrow MSCs. Proc Natl Acad Sci U S A.

[15] Kim SM, Oh JH, Park SA, Ryu CH, Lim JY, Kim DS, Chang JW, Oh W, Jeun SS (2010). Irradiation enhances the tumor tropism and therapeutic potential of tumor necrosis factor-related apoptosis-inducing ligand-secreting human umbilical cord blood-derived mesenchymal stem cells in glioma therapy. Stem Cells.

[16] Cojocneanu Petric R, Braicu C, Raduly L, Zanoaga O, Dragos N, Monroig P, Dumitrascu D, Berindan-Neagoe I (2015). Phytochemicals modulate carcinogenic signaling pathways in breast and hormone-related cancers. Onco Targets Ther.

[17] Tran TH, Mattheolabakis G, Aldawsari H, Amiji M (2015). Exosomes as nanocarriers for immunotherapy of cancer and inflammatory diseases. Clin Immunol.

[18] Hoshino A, Costa-Silva B, Shen TL, Rodrigues G, Hashimoto A, Tesic Mark M, Molina H, Kohsaka S, Di Giannatale A, Ceder S, et al. Tumour exosome integrins determine organotropic metastasis. Nature. 2015; 10.1038/nature15756 PMC4788391 26524530

[19] Peinado H, Aleckovic M, Lavotshkin S, Matei I, Costa-Silva B, Moreno-Bueno G, Hergueta-Redondo M, Williams C, Garcia-Santos G, Ghajar C (2012). Melanoma exosomes educate bone marrow progenitor cells toward a pro-metastatic phenotype through MET. Nat Med.

[20] Shin JW, Son JY, Raghavendran HR, Chung WK, Kim HG, Park HJ, Jang SS, Son CG (2011). High-dose ionizing radiation-induced hematotoxicity and metastasis in mice model. Clinical & Experimental Metastasis.

[21] Klein D, Schmetter A, Imsak R, Wirsdorfer F, Unger K, Jastrow H, Stuschke M, Jendrossek V (2016). Therapy with multipotent mesenchymal stromal cells protects lungs from radiation-induced injury and reduces the risk of lung metastasis. Antioxid Redox Signal.

[22] de Araujo FV, O'Valle F, Lerma BA, Ruiz de Almodovar C, Lopez-Penalver JJ, Nieto a, Santos a, Fernandez BI, Guerra-Librero a, Ruiz-Ruiz MC (2015). human mesenchymal stem cells enhance the systemic effects of radiotherapy. Oncotarget.

[23] Gomez-Millan J, Katz IS, Farias Vde A, Linares-Fernandez JL, Lopez-Penalver J, Ortiz-Ferron G, Ruiz-Ruiz C, Oliver FJ, Ruiz de Almodovar JM (2012). the importance of bystander effects in radiation therapy in melanoma skin-cancer cells and umbilical-cord stromal stem cells. Radiother Oncol..

[24] Farias VA, Lopez-Penalver JJ, Sires-Campos J, Lopez-Ramon MV, Moreno-Castilla C, Oliver FJ, Ruiz de Almodovar JM (2013). growth and spontaneous differentiation of umbilical-cord stromal stem cells on activated carbon cloth. J Mater Chem B.

[25] Farias VA, Linares-Fernandez JL, Penalver JL, Paya Colmenero JA, Ferron GO, Duran EL, Fernandez RM, Olivares EG, O'Valle F, Puertas A (2011). Human umbilical cord stromal stem cell express CD10 and exert contractile properties. Placenta.

[26] Siles E, Villalobos M, Valenzuela MT, Nunez MI, Gordon A, McMillan TJ, Pedraza V, Ruiz de Almodovar JM (1996). relationship between p53 status and radiosensitivity in human tumour cell lines. Br J Cancer.

[27] van der Vlist EJ, Nolte-'t Hoen EN, Stoorvogel W, Arkesteijn GJ, Wauben MH (2012). Fluorescent labeling of nano-sized vesicles released by cells and subsequent quantitative and qualitative analysis by high-resolution flow cytometry. Nat Protoc.

[28] Maitland ML, Schwartz LH, Ratain MJ (2013). Time to tumor growth: a model end point and new metric system for oncology clinical trials. J Clin Oncol..

[29] Claret L, Gupta M, Han K, Joshi A, Sarapa N, He J, Powell B, Bruno R (2013). Evaluation of tumor-size response metrics to predict overall survival in western and Chinese patients with first-line metastatic colorectal cancer. J Clin Oncol..

[30] Hanahan D, Weinberg RA (2011). Hallmarks of cancer: the next generation. Cell.

[31] Steel GG (1967). Cell loss as a factor in the growth rate of human tumours. Eur J Cancer.

[32] Valeriote F, Lin H (1975). Synergistic interaction of anticancer agents: a cellular perspective. Cancer Chemother Rep.

[33] Thery C, Regnault A, Garin J, Wolfers J, Zitvogel L, Ricciardi-Castagnoli P, Raposo G, Amigorena S (1999). Molecular characterization of dendritic cell-derived exosomes. Selective accumulation of the heat shock protein hsc73. J Cell Biol.

[34] Villalobos M, Olea N, Brotons JA, Olea-Serrano MF, Ruiz de Almodovar JM, Pedraza V (1995). the E-screen assay: a comparison of different MCF7 cell stocks. Environ Health Perspect.

[35] Haynesworth SE, Baber MA, Caplan AI (1996). Cytokine expression by human marrow-derived mesenchymal progenitor cells in vitro: effects of dexamethasone and IL-1 alpha. J Cell Physiol.

[36] Nicolay NH, Liang YY, Perez RL, Bostel T, Trinh T, Sisombath S, Weber KJ, Ho AD, Debus J, Saffrich R, Huber PE (2015). Mesenchymal stem cells are resistant to carbon ion radiotherapy. Oncotarget.

[37] Loebinger MR, Janes SM (2010). Stem cells as vectors for antitumour therapy. Thorax.

[38] Loebinger MR, Sage EK, Davies D, Janes SM (2010). TRAIL-expressing mesenchymal stem cells kill the putative cancer stem cell population. Br J Cancer.

[39] Rager TM, Olson JK, Zhou Y, Wang Y, Besner GE (2016). Exosomes secreted from bone marrow-derived mesenchymal stem cells protect the intestines from experimental necrotizing enterocolitis. J Pediatr Surg.

[40] Weyd H (2016). More than just innate affairs - on the role of annexins in adaptive immunity. Biol Chem.

