# Supplementary figures and images for: Exosomes derived from mesenchymal stem cells enhance radiotherapy-induced cell death in tumor and metastatic tumor foci
Source: Mol Cancer. 2018 Aug 15;17:122. doi: 10.1186/s12943-018-0867-0 (PMC6094906; doi:10.1186/s12943-018-0867-0)

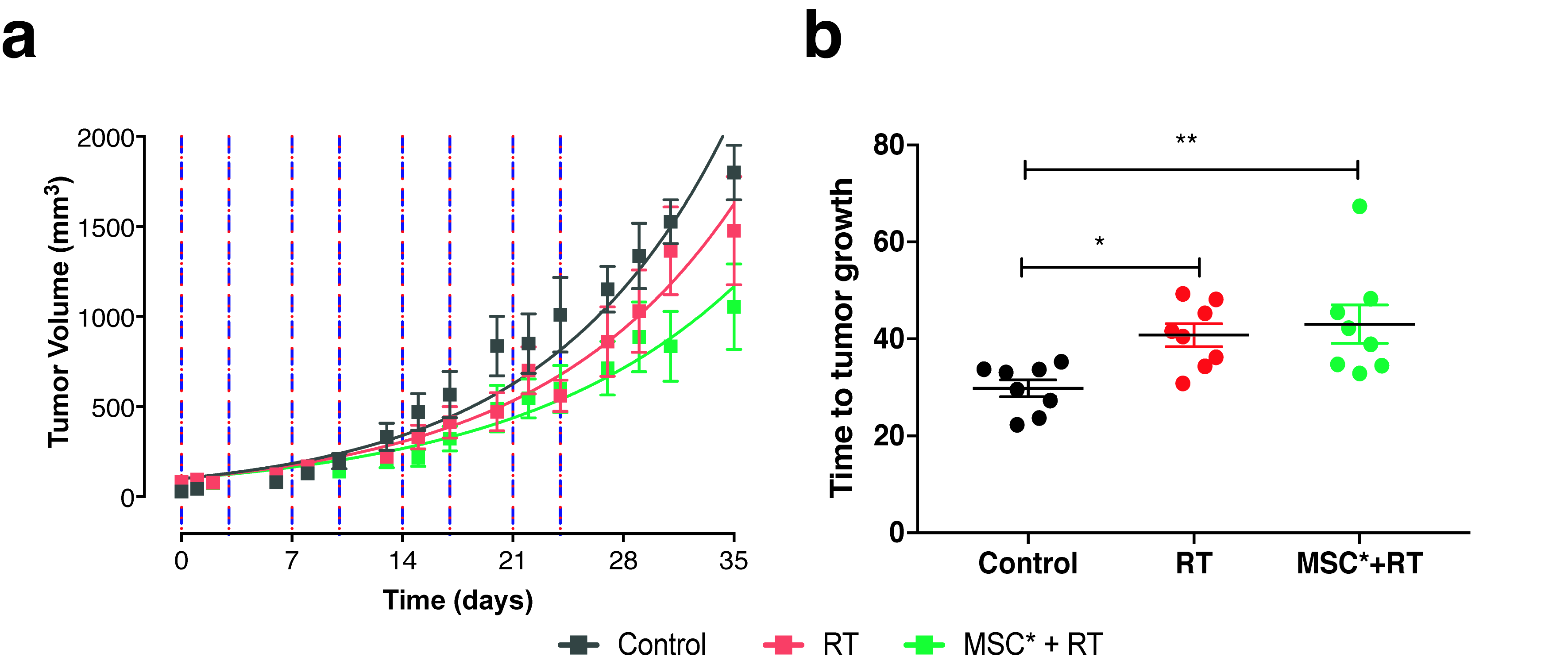

Supplement: Supplementary file 2 — Figure S1. (a) Tumor growth kinetics and response to radiotherapy administered twice-a-week alone or in combination with simultaneous MSC* injection. The combination of radiotherapy and MSC* reduced tumor growth rate more than radiotherapy alone did. (b) Calculated time to tumor growth (T-t-G) for each group. As a result of the reduction on tumor growth kinetics, tumors from the group receiving the combination of RT + MSC* would need more days to reach 2,0 ml. 3. What do the authors mean by MSC* + RT in Fig. S1. The notation (MSC* + RT) means: MSC*: in vitro activated (2 Gy of low-LET (lineal energy transfer) radiation) mesenchymal cells were administered intraperitoneally; RT: 2 h after MSC* injection tumors were treated locally with radiotherapy (RT, 2Gy). This combined treatment was repeated every 4 days during a total of 24 days. Figure S2. mRNA expression of TRAIL, DKK3 and ANXA1 by MSCs 24 and 48 h after receiving 2 Gy of radiation. The overexpression of TRAIL and DKK3 is consistent with our previous study [22], the ANXA1 overexpression is consistent with the presence of the protein form inside the MSC* exosomes. (ZIP 1301 kb) [file 12943_2018_867_MOESM2_ESM.zip › Figure_s1.tif]

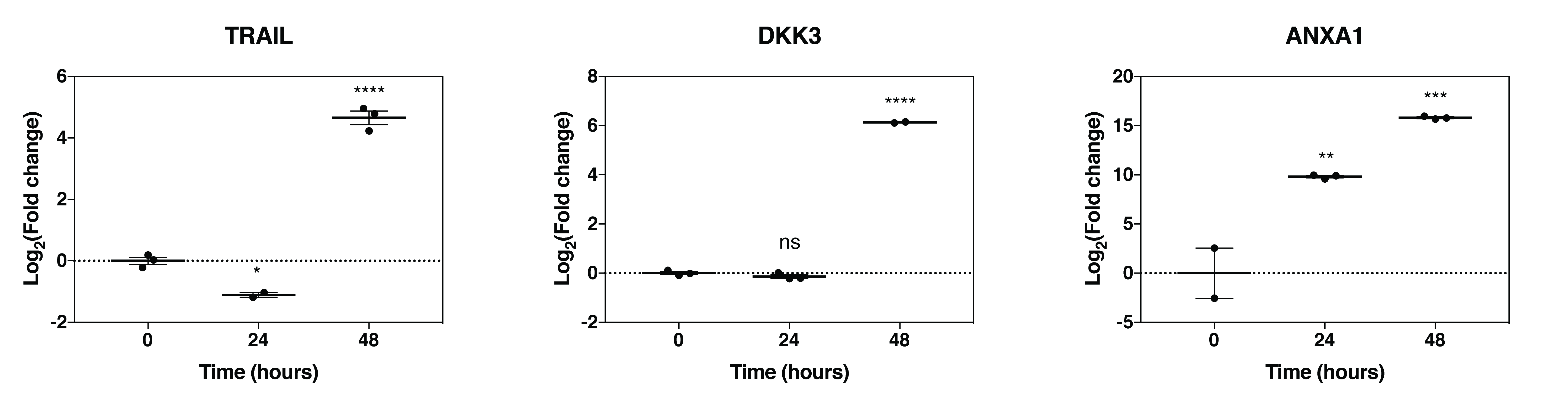

Supplement: Supplementary file 2 — Figure S1. (a) Tumor growth kinetics and response to radiotherapy administered twice-a-week alone or in combination with simultaneous MSC* injection. The combination of radiotherapy and MSC* reduced tumor growth rate more than radiotherapy alone did. (b) Calculated time to tumor growth (T-t-G) for each group. As a result of the reduction on tumor growth kinetics, tumors from the group receiving the combination of RT + MSC* would need more days to reach 2,0 ml. 3. What do the authors mean by MSC* + RT in Fig. S1. The notation (MSC* + RT) means: MSC*: in vitro activated (2 Gy of low-LET (lineal energy transfer) radiation) mesenchymal cells were administered intraperitoneally; RT: 2 h after MSC* injection tumors were treated locally with radiotherapy (RT, 2Gy). This combined treatment was repeated every 4 days during a total of 24 days. Figure S2. mRNA expression of TRAIL, DKK3 and ANXA1 by MSCs 24 and 48 h after receiving 2 Gy of radiation. The overexpression of TRAIL and DKK3 is consistent with our previous study [22], the ANXA1 overexpression is consistent with the presence of the protein form inside the MSC* exosomes. (ZIP 1301 kb) [file 12943_2018_867_MOESM2_ESM.zip › Figure_s2.tif]
